# Long-term radiographic outcomes following containment surgery for Legg–Calvé–Perthes disease in the reossification stage

**DOI:** 10.3389/fped.2026.1826534

**Published:** 2026-04-30

**Authors:** Huadong Zhang, Boxiang Li, Zhe Fu, Jianping Yang, Jianwei Hu, Shijie Liao, Zhongli Zhang

**Affiliations:** 1 Department of Pediatric Orthopedics, Tianjin Hospital, Tianjin, China; 2 Department of Trauma Orthopedics and Hand Surgery, The First Affiliated Hospital of Guangxi Medical University, Guangxi, Nanning, China

**Keywords:** containment surgery, Legg–Calvé–Perthes disease, long-term outcomes, reossification stage, therapy

## Abstract

**Background:**

This study aimed to evaluate the long-term radiographic outcomes of containment surgery in children with Legg–Calvé–Perthes disease (LCPD) during the reossification stage.

**Methods:**

This retrospective study included 58 children diagnosed with Waldenström stage III LCPD. Patients were divided into a non-surgical group (*n* = 23, mean age 8.56 ± 1.74 years) and a surgical group (*n* = 35, mean age 8.28 ± 1.29 years). All patients were monitored with serial anteroposterior pelvic and frog-leg lateral radiographs. At the final follow-up, femoral head morphology was assessed using the modified Stulberg classification, while acetabular development was evaluated using the modified Tönnis angle and the acetabular head index (AHI).

**Results:**

All 58 patients were followed until skeletal maturity. The mean follow-up duration was 7.15 ± 2.68 years in the non-surgical group and 11.94 ± 5.31 years in the surgical group. At final follow-up, in the surgical group (*n* = 35), the simplified three-group modified Stulberg classification showed 34.3% Class I (Excellent; Stulberg I–II), 51.4% Class II (Good; Stulberg III), and 14.3% Class III (Poor; Stulberg IV–V). In the non-surgical group (*n* = 23), the corresponding proportions were 17.4%, 43.5%, and 39.1%, respectively. Although a trend toward more favorable outcomes was observed in the surgical group, the difference in the proportion of acceptable outcomes did not reach statistically significant (*P* = 0.057, OR = 3.857). However, the surgical group exhibited a significantly smaller modified Tönnis angle (6.12° ± 6.27° vs. 11.63° ± 5.73°, *P* = 0.002) and a higher AHI (86.98% ± 8.99% vs. 75.51% ± 6.70%, *P* < 0.001), indicating superior acetabular morphology and coverage.

**Conclusion:**

Containment surgery performed during the reossification stage of LCPD was associated with significantly better acetabular morphology and coverage and showed a trend toward improved femoral head sphericity. These findings suggest that surgical intervention may be beneficial in select patients with LCPD during this stage of the disease.

## 1 Introduction

Legg–Calvé–Perthes disease (LCPD) is an idiopathic avascular necrosis of the capital femoral epiphysis that predominantly affects boys aged 4–8 years (1). The disease follows a protracted course through distinct stages of necrosis, fragmentation, reossification, and healing. This process often culminates in significant deformities of the femoral head and acetabulum, leading to femoroacetabular incongruity. Such incongruity is a well-established precursor to premature hip osteoarthritis and functional impairment, thereby diminishing quality of life and imposing a considerable healthcare burden (2, 3). Consequently, the principal objective in managing LCPD is to promote spherical remodeling of the femoral head, thereby preventing early-onset osteoarthritis and preserving long-term hip function (4).

Treatment strategies for LCPD are broadly categorized into conservative management and surgical intervention, with the selection dependent upon patient age, disease stage, and the extent of femoral head involvement (4, 5). Early containment, whether achieved non-surgically or via operative procedures, aims to guide the remodeling of the femoral head within the acetabular mold and has demonstrated efficacy in improving outcomes when initiated during the necrotic or fragmentation stages (6, 7). However, the optimal management strategy during the reossification stage remains controversial. By this phase, the femoral head may have already sustained irreversible deformity, and the evidence regarding the long-term benefits of surgical intervention is conflicting (4, 8–12). Some reports suggest that procedures performed during reossification may not reverse established deformities, thus limiting the potential for a favorable outcome (4). Conversely, other studies indicate that containment surgery can still yield morphological improvements, although these investigations are often limited by small cohorts and limited follow-up (8–12). Recent studies have suggested that the reossification stage is not merely a static healing phase, as femoral head deformity may continue to evolve because of physeal injury and asymmetric growth (13, 14). Furthermore, the evolution of acetabular morphology, a critical determinant of long-term prognosis, has been less frequently addressed, particularly in the context of late-stage intervention. Femoroacetabular incongruity is a primary driver of degenerative change, making acetabular development an equally important outcome measure (15–17).

Therefore, this study aimed to compare the long-term outcomes of conservative versus surgical treatment in a cohort of patients presenting with Waldenström stage III LCPD. We hypothesized that surgical containment would be associated with improved femoral head sphericity and, critically, with more favorable acetabular morphology at skeletal maturity. This investigation sought to elucidate the potential of surgical intervention to positively influence both femoral and acetabular remodeling during the reparative phase of LCPD.

## 2 Materials and methods

### 2.1 Patient selection

All patients were treated at the Department of Pediatric Orthopedics, Tianjin Hospital (Tianjin, China). All patient guardians provided informed consent, and the study received institutional review board approval. Inclusion criteria for this retrospective cohort study were: (1) Children diagnosed with Perthes disease based on radiographic findings (anteroposterior pelvic and frog-leg lateral views), aged >7 years and <12 years at diagnosis (18, 19); (2) Disease classified as the reossification stage (Waldenström stage III) on initial radiographs; (3) Radiographic evidence of skeletal maturity at final follow-up; (4) Informed consent obtained from legal guardians.

Exclusion criteria: (1) Bilateral involvement; (2) Coexisting osteochondral disorders or metabolic syndromes known to affect bone metabolism (20); (3) secondary causes of femoral head avascular necrosis (e.g., developmental dysplasia of the hip, trauma, or infection); (4) Incomplete clinical or radiographic data, or follow-up duration less than 2 years after skeletal maturity.

### 2.2 Treatment methods

Patients in the surgical group were treated with surgical containment. The decision to undergo surgery was made jointly by the treating surgeon and the patient's family. The choice of osteotomy was left to the discretion of the treating surgeon. Although these procedures differ in indication and technique, they were all categorized as containment procedures because their shared therapeutic goal was to improve femoral head containment during the reossification stage (4, 21). Postoperatively, patients were immobilized in a spica cast for 8 weeks, followed by protected weight-bearing with crutches upon cast removal. Patients in the non-surgical group were managed with abduction-internal rotation casting for 8 weeks, followed by an abduction brace. Full weight-bearing was permitted once the disease advanced to stage III b. All patients underwent follow-up with anteroposterior pelvic and frog-leg lateral radiographs every 6 months, beginning 2 months after treatment initiation. After reaching stage IV, radiographic follow-up was extended to every 2–5 years.

### 2.3 Outcome measures and radiographic assessment

**Figure 1:**
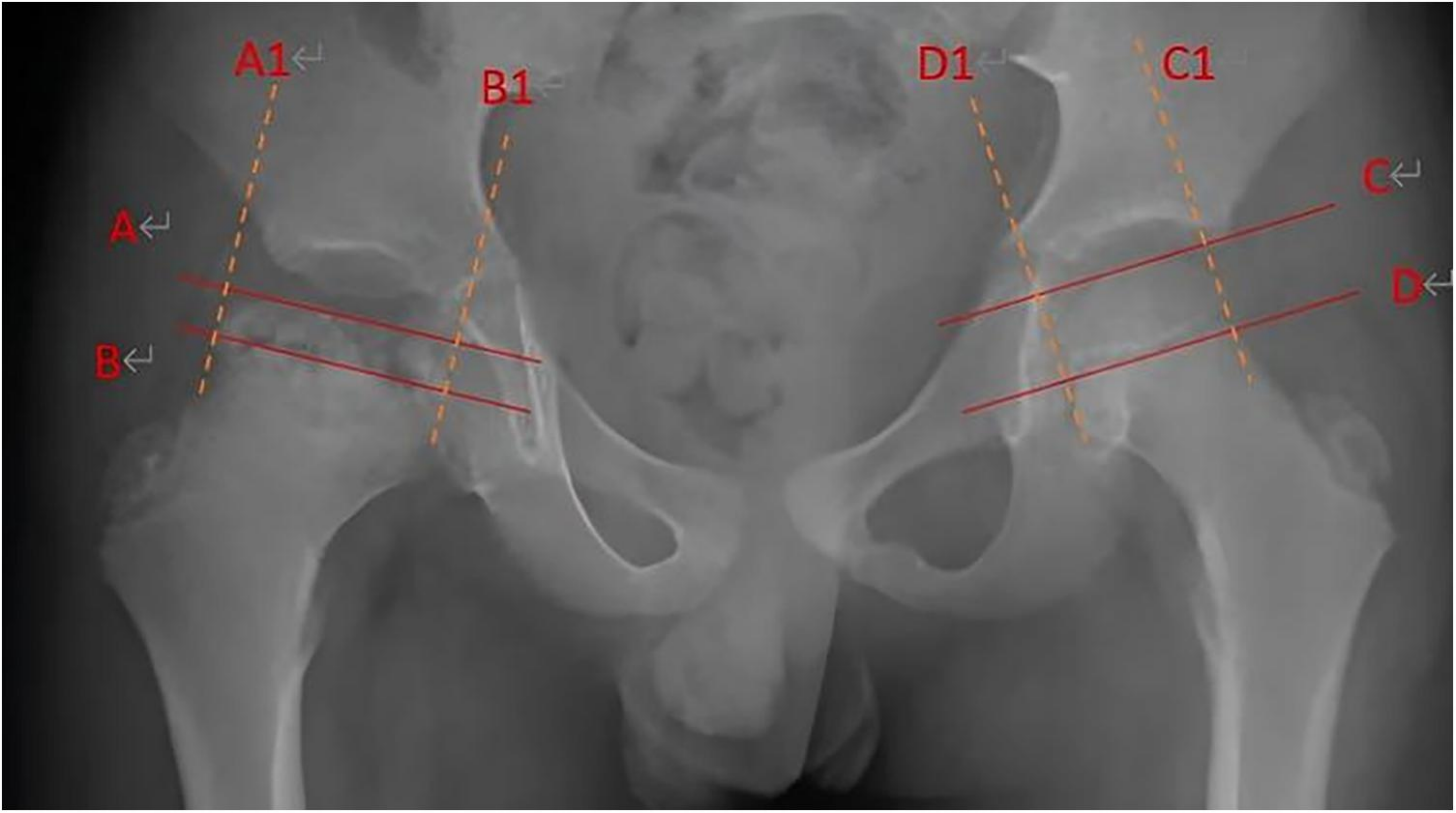
Femoral head width ratio and femoral head height ratio. Width ratio of the affected femoral head to the contralateral side = (A1B1/C1D1) × 100%. Height ratio of the affected femoral head to the contralateral side = (AB/CD) × 100%.

At the initial presentation, the following radiographic parameters were recorded to characterize the disease severity: (1) Epiphyseal Extrusion Index: To assess the degree of femoral head subluxation, the extrusion index was calculated as the percentage of the femoral head epiphysis located lateral to the Perkin's line. (2) Femoral Head Width Ratio: To quantify the broadening of the femoral head (coxa magna), this ratio was calculated by dividing the maximum width of the affected femoral head by the maximum width of the contralateral, unaffected femoral head. (3) Femoral Head Height Ratio: To quantify the degree of epiphyseal collapse, this ratio was calculated by dividing the maximum height of the affected femoral head epiphysis (measured from the superior-most point of the epiphysis to the physis) by that of the contralateral side. Both ratios were expressed as a percentage (Figure 1).

At final follow-up, femoral head morphology was evaluated using the modified Stulberg classification, with types I–III considered acceptable outcomes and types IV–V considered unacceptable (22). For descriptive analysis, the results were further condensed into three categories based on femoral head shape, as previously reported (23), because this simplified grouping has been shown to improve inter-observer reliability compared with the original five-grade Stulberg system. In the present study, these categories were descriptively defined as Class I (Excellent), corresponding to Stulberg grades I and II and representing a spherical femoral head; Class II (Good), corresponding to Stulberg grade III and representing an ovoid but congruent femoral head; and Class III (Poor), corresponding to Stulberg grades IV and V and representing a flat femoral head with an unfavorable morphology.

Additionally, acetabular morphology was assessed using the modified Tönnis angle (24) and the acetabular head index (AHI) (25). All radiographic measurements were independently performed by four experienced pediatric orthopedic surgeons, and interobserver agreement was excellent.

All patients were followed until skeletal maturity, which was selected as the primary endpoint because it is a biologically meaningful and widely accepted time point for assessing the final radiographic outcome in LCPD (4, 26).

### 2.4 Statistical analysis

Statistical analyses were performed using SPSS version 20.0 (SPSS Inc., USA). For continuous variables, independent sample *t*-tests were applied. For categorical data, the chi-square test or Fisher's exact test was used. A two-sided *p*-value of <0.05 was considered statistically significant. Weighted kappa statistics were used to determine interobserver reliability for categorical radiographic variables, including the modified Stulberg classification and the modified Waldenström stage. Intraclass correlation coefficients (ICCs) were calculated to assess interobserver reliability for continuous radiographic variables, including the epiphyseal extrusion index, femoral head width ratio, femoral head height ratio, modified Tönnis angle, and acetabular head index (AHI). Because of the retrospective design, the limited sample size, and the imbalance in follow-up duration between groups, multivariable regression analysis was not performed, as adjustment under these conditions might result in model instability and overfitting.

## 3 Results

### 3.1 Patient characteristics

A total of 58 children meeting the eligibility criteria were included in the study. All patient guardians provided informed consent, and the study received institutional review board approval. The non-surgical group comprised 23 patients (20 boys, 3 girls) with a mean age at diagnosis of 8.56 ± 1.74 years. The surgical group consisted of 35 patients (29 boys, 6 girls) with a mean age at diagnosis of 8.28 ± 1.29 years. The two groups were comparable in terms of age, sex, and laterality of involvement. The mean follow-up duration was 7.15 ± 2.68 years for the non-surgical group and 11.94 ± 5.31 years for the surgical group (Table 1).

**Table 1:** Characteristics and radiographic parameters at initial presentation.

Parameter	Non-surgical group	Surgical group	*P* value	Test statistic
Age	8.56 ± 1.74	8.28 ± 1.29	0.787	0.271
Sex (male/female)	20/3	29/6	0.732	
Side (left/right)	21/2	30/5	0.482	
Radiographic parameters				
Extrusion index (%)	29.72 ± 24.08	33.56 ± 8.64	0.472	0.729
Epiphyseal height ratio (%)	64.04 ± 16.77	57.76 ± 13.29	0.097	−1.738
Epiphyseal width ratio (%)	113.32 ± 11.92	122.68 ± 12.25	0.058	2.002
Mean follow-up duration	7.15 ± 2.68	11.94 ± 5.31	0.634	

### 3.2 Radiographic evaluation at initial presentation

#### 3.2.1 Epiphyseal extrusion index

In the non-surgical group, the femoral head epiphyseal extrusion index ranged from 10% to 63%, with a mean of 29.72 ± 24.08%. In the surgical group, the index ranged from 13% to 53%, with a mean of 33.56 ± 8.64%. There was no statistically significant difference between the two groups (*P* = 0.472, *t* = 0.729). Among the 35 hips in the surgical group, 22 were operated on during early reossification (IIIA) and 13 during late reossification (IIIB).

#### 3.2.2 Femoral head morphology

The ratio of femoral head width on the affected side to that on the contralateral side was 113.32% ± 11.92% in the non-surgical group and 122.68% ± 12.25% in the surgical group. This difference was not statistically significant (*P* = 0.058, *t* = 2.002). The ratio of femoral head height on the affected side to that on the contralateral side was 64.04% ± 16.77% in the non-surgical group and 57.76% ± 13.29% in the surgical group, with no significant difference between the groups (*P* = 0.097, *t* = –1.738) (Table 1).

### 3.3 Radiographic outcomes at final follow-up

Interobserver reliability among the four raters was evaluated for all radiographic variables. The weighted kappa value for the modified Stulberg classification was 0.83, indicating almost perfect agreement. If independently assessed, the weighted kappa value for the modified Waldenström stage was 0.86. The intraclass correlation coefficients (ICCs) for continuous radiographic variables were 0.92 for the epiphyseal extrusion index, 0.93 for the femoral head width ratio, 0.90 for the femoral head height ratio, 0.94 for the modified Tönnis angle, and 0.90 for the acetabular head index (AHI), indicating excellent interobserver reliability.

#### 3.3.1 Femoral head morphology

To evaluate the effect of surgery on femoral head sphericity, the modified Stulberg classification was used to assess radiographic outcomes at the final follow-up. In the surgical group, 12 hips (34.3%) were classified as Class I (Excellent), 18 hips (51.4%) as Class II (Good), and 5 hips (14.3%) as Class III (Poor). In contrast, the non-surgical group had 4 hips (17.4%) classified as Class I, 10 hips (43.5%) as Class II, and 9 hips (39.1%) as Class III. The distribution of outcomes at the final follow-up is detailed in Table 2. Although a strong trend toward a better outcome was observed in the surgical cohort, this difference did not reach statistical significance (*P* = 0.057; OR = 3.857). Therefore, the present data do not demonstrate a statistically significant improvement in femoral head morphology, but they suggest a possible radiographic advantage of surgical treatment. Specifically, the surgical group had a markedly lower rate of poor outcomes (Class III: 14.3% vs. 39.1%).

**Table 2:** Radiographic evaluation at final follow-up.

Outcome measure	Non-surgical group	Surgical group	*P* value	Test statistic	Effect size
Stulberg classification			0.057*	–	OR = 3.857
I, II and III	14	30			
IV and V	9	5			
Acceptable outcome rate	60.87%	85.71%			
Modified Tönnis angle (°)	11.63 ± 5.73	6.12 ± 6.27	0.002	−3.313	–
AHI (%)	75.51% ± 6.7	86.98% ± 8.99	0.001	5.369	–

* Fisher exact test.

#### 3.3.2 Acetabular morphology

To assess the effect of surgery on acetabular morphology, the modified Tönnis angle and acetabular head index (AHI) were measured on the affected side. The modified Tönnis angle in the non-surgical group was 11.63° ± 5.73° (range: 3.17–22.2°), while in the surgical group it was 6.12° ± 6.27° (range: –6.77–18.5°), showing a statistically significant difference (*P* = 0.002, *t* = –3.313). The AHI in the non-surgical group was 75.51% ± 6.70% (range: 64%–83.25%), compared to 86.98% ± 8.99% (range: 62%–100%) in the surgical group, also demonstrating a significant difference (*P* < 0.001, *t* = 5.369) (Table 2).

Further analysis of bilateral acetabular morphology was conducted within each group. For the modified Tönnis angle, results showed that in the non-surgical group, the angle on the unaffected side was 3.98° ± 3.81° (range: –3.21 to 9.65°), while on the affected side it was 11.63° ± 5.73° (range: 3.17–22.2°). In the surgical group, the modified Tönnis angle was 5.20° ± 3.03° (range: 0–11.66°) on the unaffected side and 6.12° ± 6.27° (range: –6.77 to 18.5°) on the affected side. The difference between the affected and unaffected sides in the surgical group was not statistically significant (*P* = 0.486, *t* = 0.706), indicating that surgery may have corrected the pathological increase in the Tönnis angle. For the acetabular head index (AHI), in the non-surgical group, the AHI on the unaffected side was 86.18% ± 5.81% (range: 74.58%–99.80%), compared to 75.51% ± 6.70% (range: 64%–83.25%) on the affected side, showing a statistically significant difference (*P* < 0.001, *t* = –7.553). In the surgical group, the AHI was 81.39% ± 16.22% (range: 72.00%–97.47%) on the unaffected side and 86.98% ± 8.99% (range: 62%–100%) on the affected side, with no significant difference between sides (*P* = 0.137, *t* = 1.528). These findings further suggest that surgical intervention effectively improves the AHI and corrects acetabular dysplasia in Perthes disease (Table 3).

**Table 3:** Radiographic outcomes at final follow-up.

Category	Unaffected side	Affected side	*P* value	Test statistic
Modified Tönnis angle (°)				
Surgical group	5.20 ± 3.03	6.12 ± 6.27	0.486	0.706
Non-surgical group	3.98 ± 3.81	11.63 ± 5.73	0.001	6.922
AHI (%)				
Surgical group	81.39% ± 16.22	86.98% ± 8.99	0.137	1.528
Non-surgical group	86.18% ± 5.81	75.51% ± 6.7	0.001	−7.553

**Figure 2:**
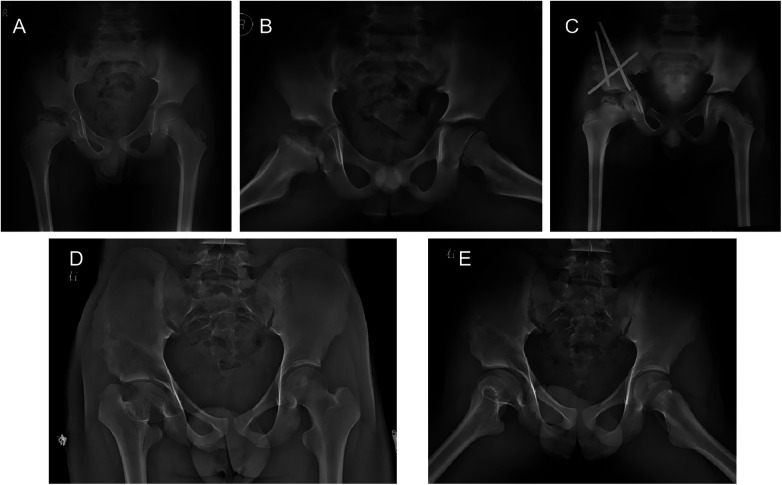
Serial radiographs of a 7-year-10-month-old boy with right femoral head necrosis in the early reossification stage treated with containment surgery. **(A)** Initial anteroposterior pelvic radiograph showing the lesion before treatment; the affected hip was the right hip. **(B)** Preoperative frog-leg lateral radiograph demonstrating loss of containment of the femoral head on the right side. **(C)** Early postoperative radiograph of the right hip after containment surgery. **(D)** Follow-up radiograph showing continued healing and remodeling of the right hip. **(E)** Radiograph at the final follow-up of 6 years and 9 months, showing the outcome of the right hip, which was classified as Stulberg grade III (class II).

**Figure 3:**
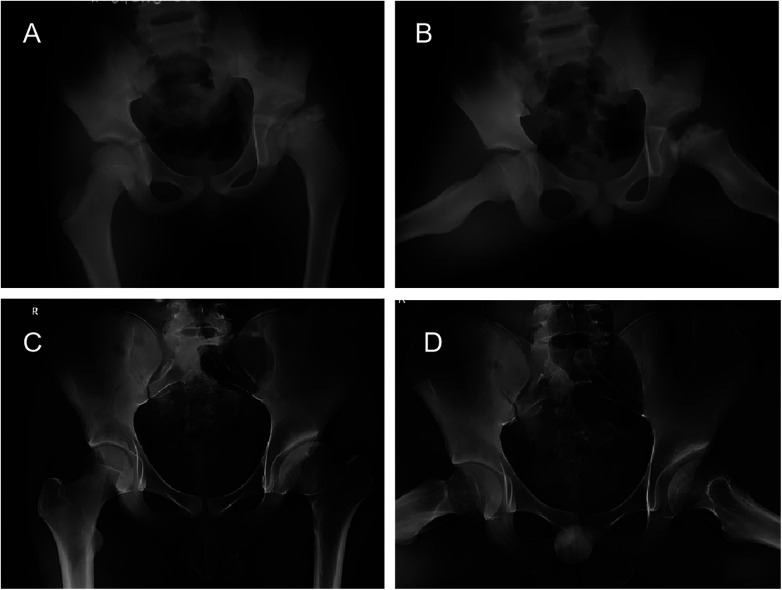
Serial radiographs of a 7-year-4-month-old boy with left femoral head necrosis in the early reossification stage who received conservative treatment. **(A)** Anteroposterior pelvic radiograph at initial presentation; the affected hip was the left hip. **(B)** Frog-leg lateral radiograph at initial presentation of the left hip. **(C)** Later follow-up radiograph showing persistent deformity of the left femoral head and acetabular changes. **(D)** Radiograph at the final follow-up of 8 years, showing that the acetabular inclination angle on the affected (left) side was markedly greater than that on the contralateral side; the outcome was classified as Stulberg grade III (class II).

Representative cases from the study are presented in Figures 2, 3.

## 4 Discussion

The principal objective in managing LCPD is to prevent femoral head deformity and achieve hip congruency to mitigate the risk of premature osteoarthritis (3, 4, 27). While most research has focused on early-stage disease, the optimal treatment for patients in the reossification stage remains an area of clinical uncertainty, with limited data on acetabular outcomes (28, 29). In this long-term follow-up study, we found that although containment surgery did not produce a statistically significant improvement in final Stulberg classification, a clear trend toward better femoral head morphology was evident (85.7% vs. 60.9% acceptable outcomes; *P* = 0.057). More importantly, surgical intervention was associated with significantly superior acetabular morphology, as demonstrated by a normalized Tönnis angle and an improved AHI. This highlights the positive influence of late-stage containment on acetabular development, a finding with important implications for long-term joint health.

The Stulberg classification remains a cornerstone for prognosticating long-term outcomes in LCPD. By employing a three-tiered system (Excellent, Good, Poor), we were able to conduct a more nuanced analysis of the outcomes. In this study, although there was no statistically significant difference in the final Stulberg outcomes between the non-surgical and surgical groups (*P* = 0.057), the higher acceptable outcome rate in the surgical group (30 hips, 85.71%) compared to the non-surgical group (14 hips, 60.87%), with an odds ratio (OR) of 3.857, suggests that patients in the reossification stage may still benefit from surgical intervention. These findings are consistent with with those of Aydin BK (28), which reported that the development of osteoarthritis and Stulberg classification outcomes were not significantly associated with the disease stage at the time of surgery (based on the Waldenström classification, *P* = 0.2). However, that study focused only on proximal femoral varus osteotomy and did not address secondary acetabular changes. In contrast, the results differ from those of M. Kadhim (30), whose study showed that only 69% of patients achieved good outcomes following late-stage surgery. However, their definition of “late-stage” included patients in both stage III and stage IV of the Waldenström classification. As the femoral head is typically fully remodeled by stage IV, it has limited surgical plasticity, thereby reducing the effectiveness of containment procedures.

In this context, the findings of Abril et al. are particularly relevant. They reported that approximately one-third of hips with LCPD may undergo an ellipsoidal or ovalization process, largely related to physeal injury and asymmetric growth, and that these hips tend to progress to less favorable spherical outcomes. In a subsequent study, Abril et al. further showed that growth modulation may improve femoral head sphericity in hips with early signs of ovalization, suggesting that femoral head shape may still be modifiable during the later phases of disease, although not uniformly so (13, 14). Therefore, the relatively high proportion of acceptable outcomes in our surgical group should be interpreted cautiously and may partly reflect patient selection, as well as the qualitative and observer-dependent nature of the Stulberg classification. This study was designed to investigate whether, even during the reossification stage when femoral head deformity is already present, surgical intervention to improve containment can promote subsequent remodeling of the femoral head under conditions of enhanced coverage, thereby improving its final morphology. Additionally, compared to previous studies, the strength of this study lies in its longer follow-up duration, allowing for a more accurate assessment of long-term outcomes. Overall, our findings support that surgical intervention during the reossification stage can optimize femoral head morphology and has meaningful clinical value. Although the operative group included different surgical procedures, these techniques generally share the common therapeutic aim of improving femoral head containment during the reossification stage (4, 5, 21, 31). Accordingly, the present findings are best interpreted as reflecting the radiographic outcomes of containment surgery as a treatment strategy, rather than the effect of any specific procedure.

A key finding of our study is the significant positive impact of surgery on acetabular morphology. It is widely accepted that acetabular changes in LCPD are secondary to femoral head deformity. Therefore, the goal of treating Perthes disease is not only to achieve a spherical femoral head, but also to ensure adequate acetabular coverage in order to prevent early-onset osteoarthritis (32). This study emphasizes the importance of acetabular morphology in the long-term prognosis of Perthes disease. Evaluation indicators included the modified Tönnis angle and the acetabular head index (AHI). In our study, the mean modified Tönnis angle in the surgical group was 6.12° ± 6.27°, significantly lower than that of the non-surgical group, which was 11.63° ± 5.73° (*P* = 0.002, *t* = –3.313). Additionally, there was no significant difference between the affected and unaffected sides in the surgical group (*P* = 0.486), indicating that containment surgery successfully reversed acetabular inclination. Shah et al. (28) found that in surgically treated hips, acetabular inclination (Sharp's angle) continued to improve from the healing stage to skeletal maturity (healing stage: 44.26° ± 3.14°, skeletal maturity: 41.26° ± 3.88°), with a statistically significant difference (*P* < 0.001, *t* = 5.928). In contrast, no significant improvement in Sharp's angle was observed in non-surgically treated hips. In a comparative study of 36 hips, M. Kamegaya (33) involving 36 hips reported no significant difference in acetabular roof obliquity between patients treated with proximal femoral varus osteotomy and those managed conservatively. These previous studies suggest that conservative treatment alone may be insufficient to prevent secondary acetabular inclination and that a femoral osteotomy alone may not correct established acetabular deformity. However, both studies primarily included patients in the early stages of disease and did not specifically focus on those in the reossification stage. In contrast, our study included only patients in the reossification stage, with follow-up extended to skeletal maturity. The results demonstrated that containment surgery significantly improved acetabular inclination at final follow-up, which may help reduce secondary acetabular steepening and femoroacetabular incongruity. A well-formed acetabulum may also contribute to improved femoral remodeling, underscoring the clinical significance of addressing acetabular morphology during treatment.

Studies have shown that poor acetabular coverage is a significant risk factor for hip osteoarthritis in the third and fourth decades of life (32). In the present study, the AHI on the affected side in the surgical group was significantly greater than that in the non-surgical group (86.98% ± 8.99% vs. 75.51% ± 6.70%, *P* < 0.001). Additionally, there was no significant difference in AHI between the affected and unaffected sides in the surgical group, whereas in the non-surgical group, the AHI on the affected side was significantly lower than that on the contralateral side. Saito et al. (32) reviewed 51 cases of Perthes disease and divided them into two groups based on the presence or absence of osteoarthritis. They found that the mean AHI in the non-osteoarthritis group (75.7% ± 9.3%) was significantly higher than that in the osteoarthritis group (66.5% ± 12.1%) (34). In our study, the postoperative AHI was significantly higher than the AHI reported in the non-osteoarthritis group in Saito's study, suggesting a potentially lower long-term risk of osteoarthritis following surgical intervention. Similarly, M. Albayrak (35) reported that at all time points, the mean AHI on the affected side was lower than that on the unaffected side, but improved significantly after surgery, reaching values not significantly different from normal. Moreover, their study showed that the reduction in acetabular coverage following disease onset could not be reversed through conservative treatment. These findings are consistent with our results and further support the advantage of containment surgery in improving acetabular coverage, which may contribute to a reduced long-term risk of osteoarthritis in patients with Perthes disease.

This study is not without limitations. First, its retrospective design introduces the possibility of selection bias, as the decision to perform surgery was based on clinical judgment rather than predefined criteria. Second, the mean follow-up duration differed substantially between the two groups, which may have influenced both femoral head remodeling and acetabular development. Third, the cohort size was relatively small, and no multivariable regression analysis was performed to adjust for potential confounders. Fourth, although interobserver reliability was formally assessed and showed excellent agreement, the study remains limited by the qualitative nature of some radiographic classifications, particularly the Stulberg system. Fifth, the study focused exclusively on radiographic outcomes, and functional measures such as pain, hip function, gait, and activity level were not consistently available. Finally, the surgical group included a variety of containment procedures (e.g., Salter, Pemberton, and Ganz osteotomies), and the potential differential effects of these specific procedures could not be analyzed because of the limited sample size. Therefore, the findings should be interpreted as reflecting the outcomes of containment surgery as a treatment strategy rather than the effect of any single surgical technique. Future research should ideally employ prospective, multicenter designs, include larger cohorts, apply multivariable modeling, and integrate functional outcomes alongside radiographic measures. Nevertheless, the strength of this study lies in its focus on a specific, controversial stage of LCPD, its long-term follow-up to skeletal maturity, and its novel emphasis on acetabular morphology as a key outcome.

## 5 Conclusion

Long-term follow-up indicates that containment surgery performed during the reossification stage of LCPD is associated with superior acetabular development and may still provide radiographic benefit when adequate containment of the femoral head within the acetabulum is achieved. By normalizing acetabular inclination and improving femoral head coverage, surgery may mitigate the long-term risk of osteoarthritis. A trend toward improved femoral head morphology was observed, although this did not reach statistical significance. These findings provide evidence to guide treatment decisions for patients with LCPD in the reossification stage and underscore the prognostic importance of acetabular morphology.

## Data Availability

The raw data supporting the conclusions of this article will be made available by the authors, without undue reservation.
